# Intraindividual Variability Measurement of Fine Manual Motor Skills in Children Using an Electronic Pegboard: Cohort Study

**DOI:** 10.2196/12434

**Published:** 2019-08-28

**Authors:** Diego Rivera, Antonio García, Jose Eugenio Ortega, Bernardo Alarcos, Kevin van der Meulen, Juan R Velasco, Cristina del Barrio

**Affiliations:** 1 Departamento de Automática Escuela Politécnica Superior Universidad de Alcalá Alcalá de Henares Spain; 2 Departamento de Psicología Biológica y de la Salud Facultad de Psicología Universidad Autónoma de Madrid Madrid Spain; 3 Departamento de Psicología Evolutiva y de la Educación Facultad de Psicología Universidad Autónoma de Madrid Madrid Spain

**Keywords:** child development, psychology, developmental, play and playthings, motor skills, smartphone

## Abstract

**Background:**

Pegboard tests are a powerful technique used by health and education professionals to evaluate manual dexterity and fine motor speed, both in children and adults. Using traditional pegboards in tests, the total time that, for example, a 4-year-old child needs for inserting pegs in a pegboard, with the left or right hand, can be measured. However, these measurements only allow for studying the variability among individuals, whereas no data can be obtained on the intraindividual variability in inserting and removing these pegs with one and the other hand.

**Objective:**

The aim of this research was to study the intraindividual variabilities in fine manual motor skills of 2- to 3-year-old children during playing activities, using a custom designed electronic pegboard.

**Methods:**

We have carried out a pilot study with 39 children, aged between 25 and 41 months. The children were observed while performing a task involving removing 10 pegs from 10 holes on one side and inserting them in 10 holes on the other side of a custom-designed sensor-based electronic pegboard, which has been built to be able to measure the times between peg insertions and removals.

**Results:**

A sensor-based electronic pegboard was successfully developed, enabling the collection of single movement time data. In the piloting, a lower intraindividual variability was found in children with lower placement and removal times, confirming Adolph et al’s hypothesis.

**Conclusions:**

The developed pegboard allows for studying intraindividual variability using automated wirelessly transmitted data provided by its sensors. This novel technique has been useful in studying and validating the hypothesis that children with lower movement times present lower intraindividual variability. New research is necessary to confirm these findings. Research with larger sample sizes and age ranges that include additional testing of children’s motor development level is currently in preparation.

## Introduction

### Overview

In the research on child development, especially in the field of manual dexterity and fine motor skills, professionals use scales and tests to help them in the evaluation of the child’s progress and in the detection of possible developmental delays [1,2]. These scales often involve the performance of specific activities using particular objects; among them are pegboards. A pegboard is a wooden or other material platform with holes, usually displayed along 2 parallel rows. A set of cylindrical pegs can be inserted into the holes. Depending on the scale, different measures are obtained with pegboards. For example, the time a child needs to place a particular number of pegs into the holes, withdrawing them from a container at the side of the pegboard is measured by means of a stopwatch (9-Hole Pegboard Dexterity Test). In another scale, the number of pegs that are placed in a given time lapse is counted (30 seconds in the Purdue Pegboard Test) [3].

### Background

Pegboards have a long tradition in research on manual dexterity, so much that some of them have not changed substantially since 1948, as is the case of the Purdue pegboard [4]. Over time, new pegboards have appeared with their corresponding normative scores [5], such as the Functional Dexterity Test, which has been designed specifically to evaluate the functional level of persons with impairments of the dominant or nondominant hand, or the 9-Hole Peg Test, with existing norms for both children and adults [6]. The latter pegboard has been included in the test battery *Toolbox Assessment of Neurological and Behavioral Function*, recommended for its high reliability, easy application, and low cost by the National Institutes of Health [7]. These various pegboards have been tested with children [8,9] and adults [10], and can be used to evaluate manual dexterity of persons with Parkinson disease [11], Down syndrome [12], Asperger syndrome or autism [13,14], or primary school children with writing difficulties [15].

When using these pegboards, measurement accuracy depends on the skills of the professional. In addition, it is not possible to measure the time of inserting each single peg using a manual stopwatch, and consequently only the total time per trial can be obtained, for example, the time of a trial that comprises inserting 10 pegs in a board with the right hand.

An essential quality of motor development is its variability [16], both among various subjects (interindividual variability), as well as within the same person at different test moments (intraindividual variability). Although traditional pegboards provide information of interindividual variability [17,18], they cannot provide immediate data on intraindividual variability. Trials would need to be repeated several times to test the variability among performances of the same individual, which is difficult to do with children, especially very young children.

A growing number of authors emphasize the importance of intraindividual variability in motor development [19-21] and relate it to a lack of motor control in the process of acquiring new skills [16]. This hypothesis has been confirmed in research that includes measuring the reaction times to stimuli presented to children with attention-deficit/hyperactivity disorder [22,23], as well as in various studies on drawing and writing, with both children with typical development and children with developmental coordination disorder [24].

However, there are few tools available for the measurement of this intraindividual variability nowadays. On the other hand, using sensor-embedded devices in electronic health environments is becoming a popular research topic as the aggregation and analysis of sensor information is a very promising feature for developing new prevention and management techniques for diverse health issues [25,26], and also there is much literature on the design of sensor-based tools for this purpose [27]. Specifically, some efforts have already been made in the design of devices to help professionals in the study of children’s developmental issues such as autism spectrum disorder [28,29].

### Objectives

Following this approach, we have designed a sensor-based electronic pegboard, which enables the measurement of the time taken for every single peg removal and placement during the activity. This board, as opposed to traditional pegboards, allows the accurate measurement of intraindividual manual skills by automatically determining the absence or presence of pegs each time a peg is moved. The board is based on the proposals of the *Desarrollo de juguetes inteligentes para atención temprana a niños con trastornos del desarrollo en el entorno educativo y en el hogar digital* (EDUCERE) project [30,31], which aimed to develop an ecosystem of connected toys and tools to aid professionals in the development assessment tasks. The main architecture and modules developed in the project are described in Rivera et al [32] and are the base of the specific tool proposed here.

Using this electronic pegboard, we have designed and conducted a cohort study with 2- to 3-year-old children for the evaluation of intraindividual variability when using it for removing and placing pegs. The study and its results are described and discussed in this paper.

The rest of the paper is organized as follows: the *Methods* section describes the study carried out, its initial hypothesis, the recruitment process, and a brief description of the designed tool. The results obtained in the cohort study are shown in the *Results* section, and finally, in the *Discussion* section, we discuss the results and the possible future steps to take in this research. Finally, we add some technological design details of the sensor-based pegboard in Multimedia Appendix 1.

## Methods

According to the hypothesis of Adolph et al [16], motor dexterity development will be accompanied by a decrease of intraindividual variability. Those children who are faster with the pegs will show less intraindividual variability, and this result will be reflected in medium- or high-level correlations between placement and removal times of each hand and the SDs.

To prove this hypothesis, we have designed a study which could determine the variability of movement times during a playing activity comprising the removal and placement of pegs in a pegboard. A total of 39 children were interviewed, 21 boys and 18 girls. The children were aged between 25 and 41 months (mean=34.03, SD 3.96) and attended a day care center for 0- to 3-year-olds, located at the university campus on the northern side of Madrid.

**Figure 1:**
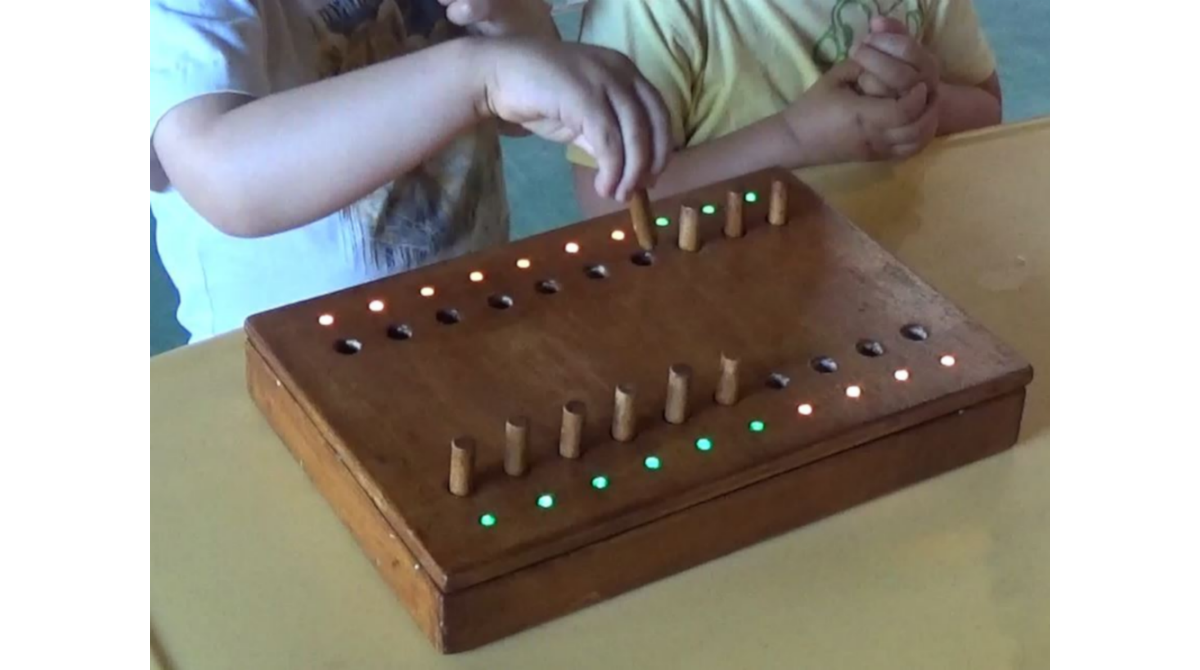
Snapshot of the prototype sensor-based pegboard being used by a child.

Once the school agreed to participate in the study, a letter was sent to the families of the 3 groups of children in the upper grade (2- to 3-year-olds), with a summary of the project and a reference to a more detailed document at their disposal in the school to obtain informed parental consent from each family (see [33] for a detailed description on ethical cautions). The project had been previously approved by the university Commission of Ethics in Science. The children were interviewed in the school setting, in a quiet room by one of the researchers, while another researcher videotaped the child-pegboard interaction and a third one supervised the registration of the activity information. Every child was invited to play with the board and move 5 of the 10 pegs, finding out how the lights would change. After this familiarization activity, the test started, and each child was invited to move the 10 pegs from their original position in row A (the row positioned facing the professional) to the empty holes in row B (the row positioned facing the child; first trial). Once finished, the child was asked to perform the same task with the other hand (second trial). A snapshot of the videotaped activity can be seen in Figure 1.

**Figure 2:**
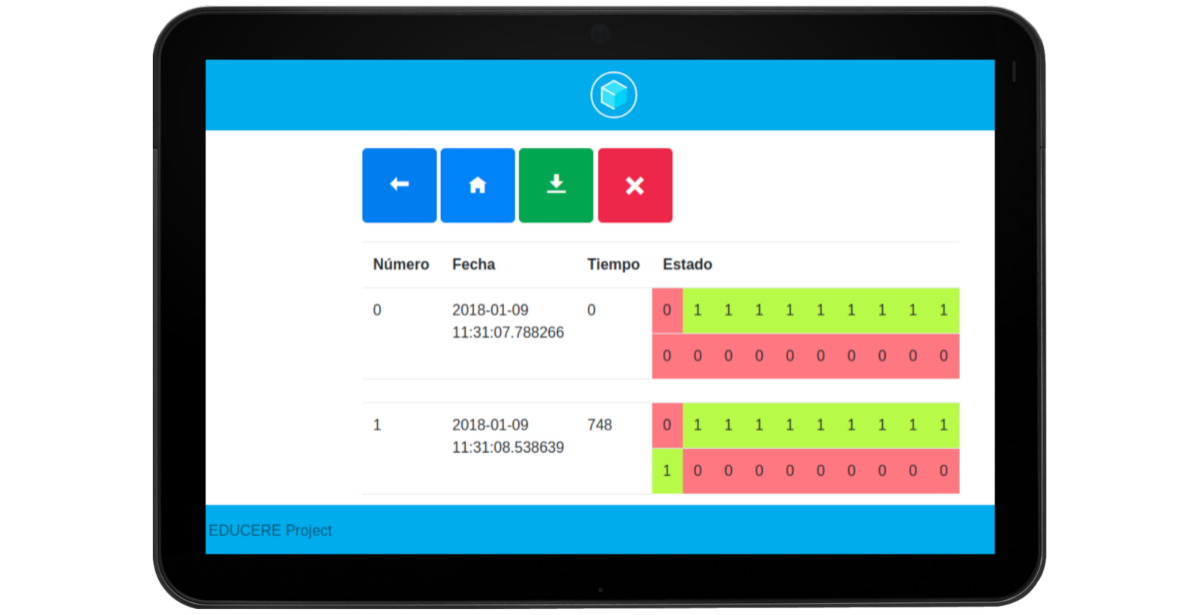
Example of results of a test as shown in the Web interface from a tablet.

Given that the traditional pegboards do not allow to measure the intraindividual variability during its use by a single child, we have designed and developed a prototype of a sensor-based electronic pegboard. The physical design of the pegboard proposed in this paper has been based on the current designs used for dexterity tests to maintain the reliability of the tests as much as possible. The design of the board is based on a series of requirements derived from (1) the experts’ needs related to the assessment tests (eg, the mobility-driven design of the device or its size), (2) the children’s interactions with this type of tools (ie, the necessity of a user-friendly simple interface and interaction without any special learning requirement for its use), and (3) the technological limitations and its costs. These requirements have been compiled from different sources such as the literature on pegboard tests, experts’ knowledge of the matter, and the documentation on the available sensor and communications technologies in these environments. Considering these requirements, the proposed electronic pegboard design has been developed as a modular system comprising the pegboard itself, a data collector module, and a user interface accessed through mobile or desktop devices (an example of this interface is shown in Figure 2).

**Figure 3:**
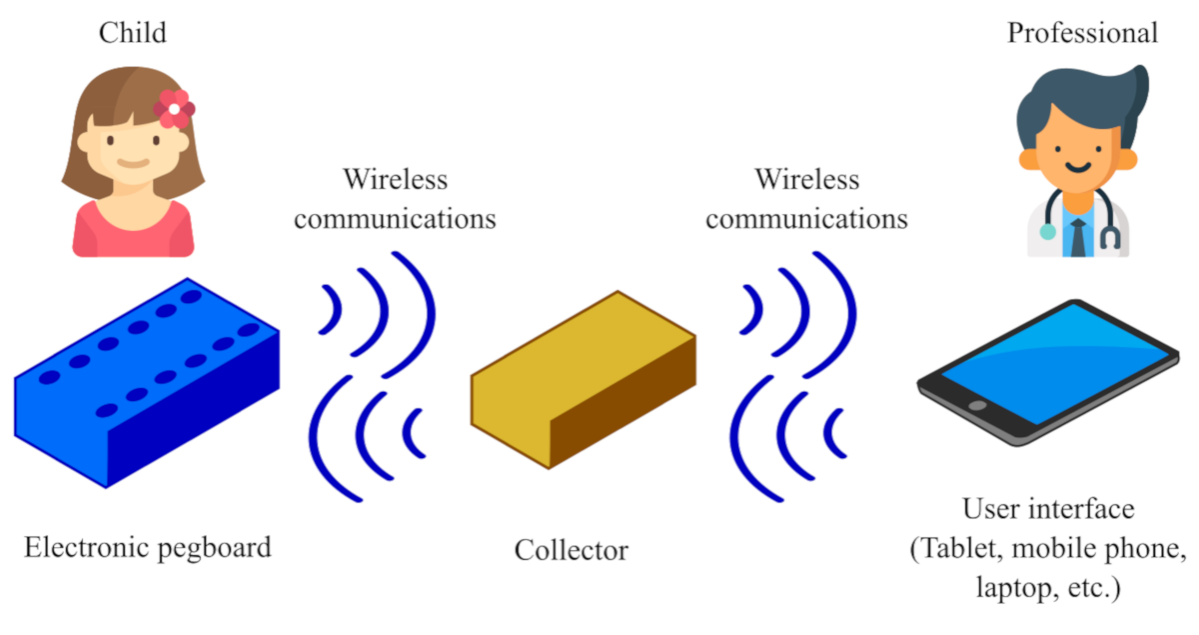
Electronic pegboard system components overview.

The modules are independent and communicate between each other using a wireless communications system. The full components overview and its communications can be seen in Figure 3. Further details on the modules’ design can be found in Multimedia Appendix 1.

To determine the reliability of the pegboard, an additional study was carried out. In this study, the total trial times of the activity of 17 children with the pegs were recorded manually by an expert in manual dexterity using a stopwatch and was compared with the total times obtained electronically. The children were aged between 30 and 41 months and completed 2 trials, one with each hand. Hereafter, the measurements were analyzed, comparing the manually registered with those using the electronic pegboard, finding a high Pearson correlation between the manually and the electronically measured times of the first (*r*=0.998; *P*<.001) and the second trials (*r*=0.997; *P*<.001).

## Results

The electronic pegboard has enabled the study of intraindividual variability as the system allows for recording 10 placement times (for each peg) and 9 removal times (for pegs 2 to 10, excluding the first one) in a single trial, instead of 1 single time measurement for the total amount of pegs. In addition, it is possible to obtain the individual profiles of task performance for each child. In the following paragraphs, results concerning the intraindividual variability and individual profiles are reported.

Specifically, we have recorded the mean and intraindividual values (IIV) for each child performing the activity. The values have been classified depending on the hand used for the movement and the type of movement (placement or removal of pegs) in 4 groups:

- right hand placement (RP)
- left hand placement (LP)
- right hand removal (RR)
- left hand removal (LR)

The IIV value for each child is calculated as the SD of the times measured during the activity for each group. These values along with the mean time taken for each movement can contribute to determine if there is a relationship between the intraindividual variability, measured as the time taken to move the pegs, and the dexterity (the mean speed of placement or removal for each hand).

A correlation analysis among age, the 4 mean measures of dexterity (mean RP, mean LP, mean RR, and mean LR), and the corresponding IIV values was performed. Results (see Table 1) show various negative correlations with age (eg, older children are faster at placing pegs with the right hand). Moreover, positive correlations between mean values and IIV for each hand and movement were found, from *r*=0.46 (*P*=.003) to *r*=0.72 (*P*<.001).

**Table 1:** Pearson correlations between age and placement and removal measures.

Measure^a^	Age	Mean^b^ RP^c^	IIV^d^ RP	Mean LP^e^	IIV LP	Mean RR^f^	IIV RR	Mean LR^g^	IIV LR
Age	—^h^	—	—	—	—	—	—	—	—
Mean RP	−0.33^i^	—	—	—	—	—	—	—	—
IIV RP	−0.17	0.66^j^	—	—	—	—	—	—	—
Mean LP	−0.31	0.44^j^	−0.01	—	—	—	—	—	—
IIV LP	−0.38^i^	0.35^i^	0.20	0.46^j^	—	—	—	—	—
Mean RR	−0.18	0.57^j^	0.19	0.24	0.13	—	—	—	—
IIV RR	−0.19	0.29	−0.01	−0.05	−0.15	0.72^j^	—	—	—
Mean LR	−0.31	0.47^j^	0.14	0.36^i^	0.27	0.52^j^	0.36^i^	—	—
IIV LR	−0.40^i^	0.35^i^	0.15	0.13	0.21	0.25	0.40^i^	0.72^j^	—

^a^All tests are 2-tailed.

^b^Mean: mean value for each movement in an activity.

^c^RP: right hand placement.

^d^IIV: intraindividual variability (SD for the movements in an activity).

^e^LP: left hand placement.

^f^RR: right hand removal.

^g^LR: left hand removal.

^h^Empty cells are meant to avoid repeated correlation values (the table is diagonally symmetric).

^i^*P*<.05.

^j^*P*<.01.

**Figure 4:**
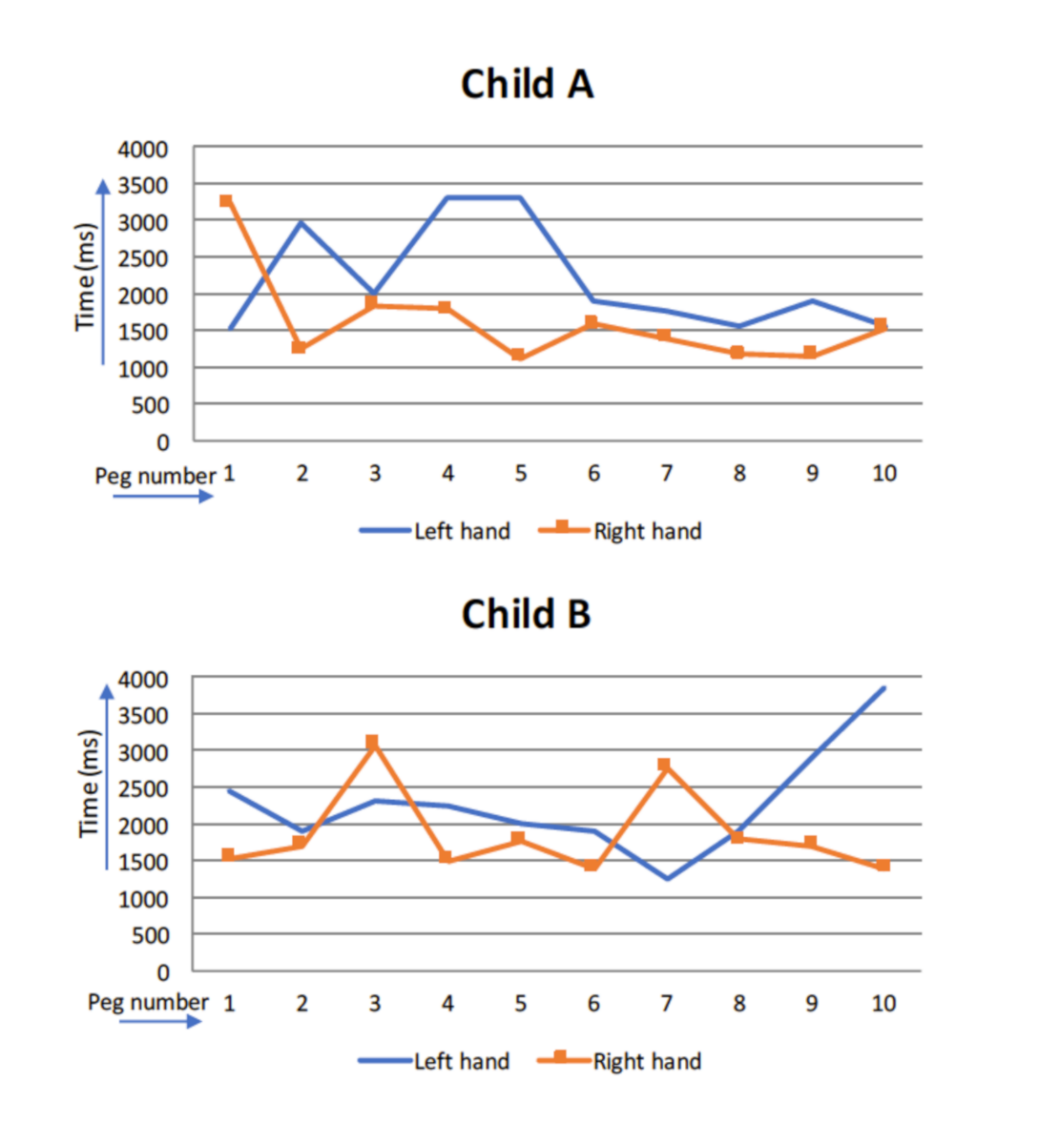
Placement times in ms with left and right hands in two children (Child A and Child B). ms: milliseconds.

The increased measurement possibilities with the electronic pegboard makes it possible to obtain individual profiles of the children showing the degree of variability in their performances [15]. As an example, Figure 4 shows placement times of the 10 pegs with both the right and the left hands of 2 children, both 30 months old. The mean time of Child A’s activity with the left hand is 2174.40 millisecond and the IIV value is 728.30 millisecond; with the right hand, mean=1418.78 millisecond and IIV=272.31 millisecond. Child B’s mean time for task performance with the left hand is 2089.78 millisecond and IIV=463.20 millisecond; with the right hand, mean=1725.00 millisecond and IIV=416.04 millisecond.

For instance, by comparing these results for the 2 children, the differences in their profiles can be observed. Child A shows a lower mean and variability of the right hand compared with the left hand, which possibly reflects the further development of a right manual preference. However, Child B displays more comparable means and very similar variabilities of both hands, and therefore no clear manual preference.

## Discussion

The pilot study carried out with children has proven to be useful to study the intraindividual variability in children. On the basis of Adolph et al’s [16] hypothesis, a lower intraindividual variability was expected in individuals with lower placement and removal times as their motor control increases. Our results show this relation in 3-year-old children, thereby confirming the initial hypothesis.

Nevertheless, some limitations can be observed in this preliminary study. A first limitation, as a consequence of its exploratory nature, is the low number of participating children. Although significant results were found, this low number of participants did now allow applying Bonferroni corrections, which is necessary when the number of correlations is high, as the results showed. Second, the age ranges of the participants were reduced, which might have caused lower correlations because of the increased homogeneity of the studied population. A third limitation lies in the absence of additional testing of the motor development level and manual dexterity of the children, as well as their manual preference, even though this may not be apparent yet. These issues are being addressed in subsequent studies.

From a technological point of view, we have presented in this paper a novel sensor-based pegboard design, which shows interesting features for the motor development in children. The study carried out can be seen also as a validation test of the tool design, as the 2- and 3-year-old children have been able to perform all the requested activities without any added difficulty derived from its design.

In future research, we will work on the integration of the pegboard within an ecosystem of similar sensor-based wireless devices that contribute to centralized data storage and analysis from different activities and perspectives, and therefore, improve the development assessment task. This would facilitate early interventions preventing children from more severe difficulties in manual activities, including handwriting. Moreover, this instrument seems promising for more precise diagnoses of eventual motor difficulties not only in children with atypical development, but in adults with Parkinson disease as well [11-15,34].

## Appendix

Multimedia Appendix 1 Sensor-based pegboard design.

## Abbreviations

EDUCERE: Desarrollo de juguetes inteligentes para atención temprana a niños con trastornos del desarrollo en el entorno educativo y en el hogar digital
IIV: intraindividual values
LP: left hand placement
LR: left hand removal
RP: right hand placement
RR: right hand removal
