# Supplementary material for: Intraindividual Variability Measurement of Fine Manual Motor Skills in Children Using an Electronic Pegboard: Cohort Study
Source: JMIR Mhealth Uhealth. 2019 Aug 28;7(8):e12434. doi: 10.2196/12434 (PMC6738021; doi:10.2196/12434)
Supplement: Multimedia Appendix 1 [file mhealth_v7i8e12434_app1.pdf]

Multimedia Appendix 1: Sensor-based pegboard design

The electronic pegboard system is based on three main modules or components that exchange information wirelessly: The pegboard itself, a collector module and a user interface.

The *pegboard* is the main module of the proposal, and it is composed of a board with 2 lines of 10 holes each, and a set of pegs that can be inserted in them. The device integrates sensors and a microcontroller (specifically an ATMega328p) that detect the insertion or removal of each peg in each hole, and a wireless communications device (A Nordic NR24L01+) that sends the data obtained from the sensors. The sensors embedded in the device allow the transmission of the whole board status, every time there is any change in one of the holes. This enables the possibility of observing the time intervals between each action of the child playing with the toy.

The *collector* is the module that manages the beginning and end of each playing activity or experiment. It sends specifically defined commands through the wireless connection to the toy, and then receives and stores the data generated by it during the test.

The commands are issued by the professionals in charge of managing the tests, using a custom *user interface* accessed through a mobile or desktop device (a tablet, a smartphone, a laptop, etc.), which is connected wirelessly to the collector. This user interface is also used to provide the necessary information about the child performing the test. This would make it possible to perform a further analysis of the gathered data. The user interface has been deployed in the collector module through a web-based application and can be accessed by any device through a web browser.

The collector has been implemented using a low-cost computer (specifically, a Raspberry Pi 3 board) with Wi-Fi capabilities. It has been configured to work as a Wi-Fi access point which can be easily connected from each mobile or desktop device located nearby.

The requirement of maintaining the pegboard as close as possible to the original designs derives from the need of guarantee that the previously tested scales for the dexterity measurements would be valid using this tool. This means that there are some constraints in terms of the object dimensions and form. Although various board designs with very different dimensions can be found in the literature, when designing a board for children usage, one needs to consider that it should be adequate for its users to play with it comfortably. For instance, the dimensions should be adapted to the arm size (in our case, the board's dimensions are 298 x 198 mm) to the child fingers (we have built 10mm pegs and 12 mm holes that a comfortable insertion) and to the desired movement range for pegs (we have set a distance of 109 mm between the center of holes of each row). Apart from the board's physical design, it has been equipped with electronic components that provide the measurement and communications capabilities. For this purpose, an Arduino-compatible microcontroller and a radiofrequency transceiver has been used. The system is powered by a Lithium Ion polymer (LiPo) battery which allows performing the testing activities in any available place without depending on a wall socket located nearby.

For the detection of peg movements into the holes, a photo interruptor sensor has been selected, that is placed under each hole inside the pegboard. This kind of sensor has a U shape and is composed by a light emitter located in front of a light receiver (see Figure 1). When the light emitter is powered, it emits a light beam that is detected by the light receiver, pulling out a low electric level in the sensor output. If a peg is placed in the

hole, the beam of light is interrupted, and the sensor output changes its status, from high level to low level.

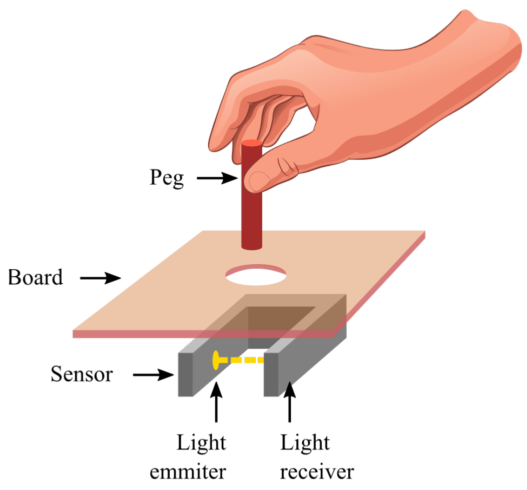

Figure 1. Photo interruptor sensor placement diagram.

Reading the status of the entire table at a given instant of time requires reading the signals offered by each of the 20 sensors placed under each hole in the board. Given that the microcontroller does not have 20 digital inputs available to read the sensors in parallel, we have included an extra circuit to read all the sensors from a single digital input in serial mode. This circuit is based on four 8 bits shift registers.
Figure 2 is a picture of the device with the board upper case removed, on which the main components of the prototype are labeled. The SPI interface is used to update the microcontroller program from a computer. The figure also shows a row of LEDs and a buzzer have been added for each row in the table. This allows for adding new interactive functionalities to the traditional pegboard (e.g. by playing a sound when an activity is finished or by modifying lights when the pegs are inserted or removed). The benefits of this simple hardware user interface will be explored in future studies.

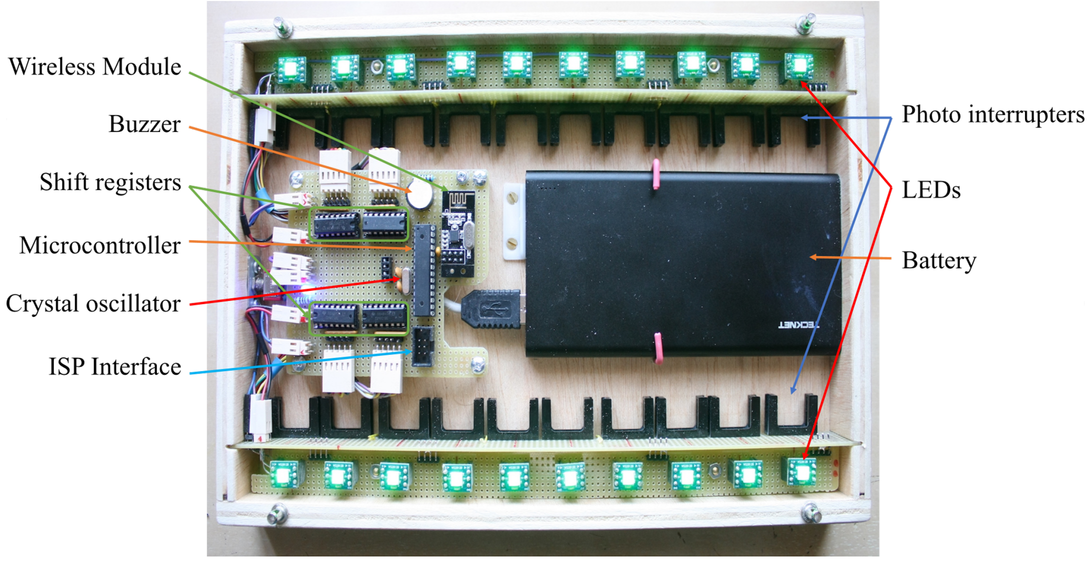

Figure 2. Details of components inside the pegboard prototype.

We have determined empirically (by comparing the measured times with an external measuring device) that the accuracy in time measurement using the pegboard is approximately ±2.5 ms, which implies an important improvement when compared to the traditional measurements, which are done manually in the tests.
The collector, on the other hand, acts as an interface between the pegboard and the professional management of the activities. It is therefore responsible for controlling the communication process with the pegboard, storing sensor data, and display them to the professional for analysis purposes. As has been mentioned before, the professional uses a Web application to control the activities of the children when using the pegboard. During a session (that is, a set of tests carried out with a group of children), when a child is ready to start the activity, the professional will send commands to the pegboard, to start and stop the test and register the sensor data. Once the session has finished, the professional is able to obtain the results from the application in the interface and download them in a CSV formatted file. The collector is configured as a wireless communications Access Point to allow wireless connections from a tablet or a computer. It also includes a radio frequency transceiver to communicate with the pegboard through the NR24L01+ module mentioned before. The collector contains a web server where the user interface application is deployed. Additionally, it serves a RESTful API to control the toy through client applications. The API is composed of various endpoints that allow the management of the activities, tests, users' information or the resultant data. This API-driven control allows for the easy development of applications and interfaces in different platforms. The main processing module in the collector is managed by a multithreaded system daemon which listens to the messages received from the pegboard and stores the information in an internal SQLite database which is also used for management information storage.

Abbreviations

API: application program interface
CSV: Comma-Separated Values
SPI: Serial Peripheral Interface
LED: light emitting diode
REST: representational state transfer
